# Correlation of the National Board of Medical Examiners Emergency Medicine Advanced Clinical Examination Given in July to Intern American Board of Emergency Medicine in-training Examination Scores: A Predictor of Performance?

**DOI:** 10.5811/westjem.2015.9.27303

**Published:** 2015-11-12

**Authors:** Katherine Hiller, Doug Franzen, Corey Heitz, Matthew Emery, Stacy Poznanski

**Affiliations:** * University of Arizona, Department of Emergency Medicine, Tucson, Arizona; † University of Washington, Department of Emergency Medicine, Seattle, Washington; ‡ Virginia Tech Carilion, Department of Emergency Medicine, Roanoke, Virginia; § Michigan State University College of Human Medicine, Department of Emergency Medicine, East Lansing, Michigan; ¶ Wright State University, Department of Emergency Medicine, Dayton, Ohio

## Abstract

**Introduction:**

There is great variation in the knowledge base of Emergency Medicine (EM) interns in July. The first objective knowledge assessment during residency does not occur until eight months later, in February, when the American Board of EM (ABEM) administers the in-training examination (ITE). In 2013, the National Board of Medical Examiners (NBME) released the EM Advanced Clinical Examination (EM-ACE), an assessment intended for fourth-year medical students. Administration of the EM-ACE to interns at the start of residency may provide an earlier opportunity to assess the new EM residents’ knowledge base. The primary objective of this study was to determine the correlation of the NBME EM-ACE, given early in residency, with the EM ITE. Secondary objectives included determination of the correlation of the United States Medical Licensing Examination (USMLE) Step 1 or 2 scores with early intern EM-ACE and ITE scores and the effect, if any, of clinical EM experience on examination correlation.

**Methods:**

This was a multi-institutional, observational study. Entering EM interns at six residencies took the EM-ACE in July 2013 and the ABEM ITE in February 2014. We collected scores for the EM-ACE and ITE, age, gender, weeks of clinical EM experience in residency prior to the ITE, and USMLE Step 1 and 2 scores. Pearson’s correlation and linear regression were performed.

**Results:**

Sixty-two interns took the EM-ACE and the ITE. The Pearson’s correlation coefficient between the ITE and the EM-ACE was 0.62. R-squared was 0.5 (adjusted 0.4). The coefficient of determination was 0.41 (95% CI [0.3–0.8]). For every increase of one in the scaled EM-ACE score, we observed a 0.4% increase in the EM in-training score. In a linear regression model using all available variables (EM-ACE, gender, age, clinical exposure to EM, and USMLE Step 1 and Step 2 scores), only the EM-ACE score was significantly associated with the ITE (p<0.05). We observed significant colinearity among the EM-ACE, ITE and USMLE scores. Gender, age and number of weeks of EM prior to the ITE had no effect on the relationship between EM-ACE and the ITE.

**Conclusion:**

Given early during intern year, the EM-ACE score showed positive correlation with ITE. Clinical EM experience prior to the in-training exam did not affect the correlation.

## INTRODUCTION

Incoming interns to emergency medicine (EM) residencies come from a variety of educational backgrounds, creating significant variations in their baseline funds of knowledge. Program directors must quickly ascertain if any interns have unusual knowledge gaps or learning difficulties that may require a specialized learning plan or remediation. Traditionally, the first high quality, objective testing available for assessment of interns has been the American Board of EM (ABEM) in-training examination (ITE), which is offered annually on the last Wednesday in February. According to the ABEM website, “It is a standardized examination that residents and program faculty can use to judge an individual resident’s progress toward successful ABEM certification. There is a strong relationship between in-training and qualifying examination scores. Physicians with higher in-training scores have a higher likelihood of passing the qualifying examination and those with lower scores have a lower likelihood of passing the qualifying examination.”^1^ This statement is supported by an observed moderate correlation between post-graduate year (PGY) 3 ITE scores and ABEM written examination scores.^2^ In addition to providing predictive information to program directors regarding the residents they are about to graduate, the ITE also provides national norms for residents at all other PGY levels of training.

United States Medical Licensing Examination (USMLE) scores also provide information for program directors about their incoming interns’ baseline knowledge. Step 1 scores are mildly correlated (R2 0.25) and Step 2 scores are moderately correlated (R2 0.43) with the EM ITE.^3^ However, USMLE Step 2 exams are typically taken in the fall of the fourth year of medical school (M4) and the ITE is not given until late winter. This gap of roughly 18 months is also one of the most variable periods in medical education in both content and clinical exposure for graduating M4s/incoming interns.^4^ Some program directors have responded to the disparity in incoming interns by providing “boot camps” to their interns immediately before, during or after orientation in July.^5^–^7^

In April 2013, the National Board of Medical Examiners (NBME) released the EM Advanced Clinical Examination (EM-ACE).^8^ This is an examination based on the national fourth year EM medical student curriculum first published in 2006 and updated in 2010.^9^,^10^ It is intended to be administered to fourth year students at the end of their EM rotation. To rapidly administer the examination and calculate scaled scores and internal validity, the NBME offered the EM-ACE free of charge for the first year of administration. In response, several residency program directors administered the EM-ACE to their incoming interns in July to identify interns who required additional educational exposure or attention. However, there is no data on whether performance on the EM-ACE, when administered to EM residents at the onset of internship, has any predictive value to known outcome measures such as ITE performance.

The objective of this study was to determine the correlation, if any, between intern scores on the EM-ACE administered in July, and intern scores on the ITE administered the subsequent February. In addition, we sought to assess whether USMLE scores correlate with EM-ACE scores when administered at the onset of internship, as USMLE scores have been shown to correlate with intern ITE scores.

## METHODS

This study was approved by the institutional review board of each participating residency, and was determined exempt from human subjects review.

This was a multi-institutional, observational study. In July 2013, entering EM interns at six geographically diverse residency programs took the EM-ACE. They underwent standard residency training, and in February 2014 took the required ABEM ITE. Scaled examination scores for both the EM-ACE and the internship ABEM ITE score were collected electronically from program coordinators and/or program directors. Additional data collected included the date of EM-ACE administration, institution, gender, age, USMLE Step 1 and Step 2 scores, and number of weeks of clinical EM and off-service experience completed during their current residency program prior to taking the ITE.

We performed linear regression to determine the relationship between EM-ACE scores and the EM ITE scores, and calculated Pearson’s correlation coefficients. Data was collected with Microsoft Excel 2007 and analyzed using StataMP-11 (College Station, TX).

## RESULTS

A total of 62 interns took the ITE at six residency programs. Of these, 60 (96.8%) also took the EM-ACE in July of their intern year. Two residents were sick on the date of EM-ACE administration, both from the same institution. Data were available for USMLE Step 1 in 50 (80.6%) and Step 2 in 48 (77.4%) of these residents. Scores on the Comprehensive Osteopathic Medical Licensing Examination (COMLEX) 2 were available for six (9.7%) of the residents. See Table 1.

Gender was slightly skewed towards male (58.06%), and the average age of the interns at examination administration was 30 years old. On average, interns had experienced 17 weeks (SD 4.4) of clinical time in the emergency department (ED) from the start of EM residency to the ITE. The average EM-ACE score was 69.8 (SD 7.1), and the average ITE score (percent correct) was 70.5% (SD 8.4%).

In a linear regression model using all the available variables, gender (p=0.99), age (p=0.52) and clinical exposure to EM prior to the ITE (p=0.53) were not associated with the in-training score. USMLE Step 1 (p=0.61) and Step 2 (p=0.53) were likewise not associated with ITE score. There were too few COMLEX scores to allow incorporation into the linear regression model.

The EM-ACE score was significantly associated with the ITE score (p<0.05). The coefficient of determination was 0.41 (95% confidence interval 0.3–0.8); in other words, for every increase of one in the scaled EM-ACE, we observed a 0.4% increase in the EM in-training score. The Pearson’s correlation coefficient between the EM ITE score and the EM-ACE was 0.62. R-squared value was 0.5 (adjusted 0.4).

In a regression model only containing the EM-ACE (the only variable significant in the full model), the significance was even higher (p<0.001, coefficient of determination 0.7 (0.4–1.0); however, the correlation was lower (R-squared 0.38). Interestingly, a similar pattern of independent association with the ITE was observed with USMLE Step 1 scores (p<0.001, R-squared 0.39) Step 2 (<0.001, R-squared 0.33) and COMLEX scores (P<0.05, R-squared 0.73) when used alone in a linear regression model predicting ITE score. In a model with all three exams (EM-ACE, Step 1 and Step 2 [there were too few COMLEX scores to include in the limited regression model]), the EM-ACE (p<0.05) and Step 1 (p<0.05) were still significantly associated with the in-training score, but Step 2 scores were not (p=0.43)

There was significant colinearity observed among the EM-ACE, the EM ITE and both USMLE Step scores (Table 2).

## DISCUSSION

To provide effective education to learners, it is important to first assess their baseline knowledge. The general nature of the medical knowledge assessed by the USMLE, as well as the variation in timing of administration, especially as related to the timing of a student’s EM rotation, makes the USMLE a less specific assessment of basic EM knowledge. Additionally, clinical experience after taking the USMLE is highly variable, ranging from one EM rotation in medical school to post-graduate experience.^4^ As a result, incoming interns may have widely different clinical EM exposure and expertise. The ABEM ITE is an excellent tool for assessment of resident knowledge, and is predictive of performance on the EM qualifying examination. Program directors have been using the ITE as a means to assess their learners’ progress towards competency since 1985.^11^ However, the date of administration is fixed, and is set eight months into a 36–48 month residency.

The EM-ACE administered at the onset of residency, halfway through the most variable 18 months of EM training, correlates well with internship ITE scores, and may provide an earlier assessment of knowledge than the in-training exam. Identifying below-average performers is of particular importance to program directors, as early identification of these learners makes early intervention with a specialized learning plan possible and allows more time for remediation. In addition to assisting program directors with identification of potential problem learners, once baseline performance on the EM-ACE is known, the ITE could then serve as an assessment of teaching methods.

Interestingly, despite the observed variation in clinical experience at the six residencies, clinical ED experience did not affect the correlation between EM-ACE and ABEM examination performance. There are a number of potential explanations for this finding. First, and most obvious, as neither the EM-ACE nor the ABEM ITE assess clinical competency, it may be that clinical experience and exposure have a much greater effect on assessment methods that are sensitive to gains in clinical competence. It may also be that programs with less clinical exposure augmented their residents’ learning by non-EM clinical activities (simulation, off-service rotations, didactics, self-learning) and vice versa. It is also possible that programs which assigned residents to less early EM-based clinical time had a greater focus on efficient learning in the limited time residents had in the ED.

## LIMITATIONS

There are a number of limitations to this study. The EM-ACE was not intended for use as a “pre-test” for internship. It was intended as a high stakes examination for fourth-year EM medical students, and is based on the national fourth-year medical student curriculum.^9^ The ITE is based on the EM model curriculum^12^_ENREF_11. While there is a large amount of overlap between these two curricula, they are not identical.

Performance on the EM-ACE (when used at the beginning of residency) is potentially affected by the weight an intern perceives a program director places on it. A lack of preparation could have negatively affected performance on the EM-ACE, as compared with a relatively augmented score on the ITE if an intern prepared more for the latter. Some of the incoming interns may have already taken one form of EM-ACE. This is unlikely, as the EM-ACE was offered from April 2013 on; however, if a student took an EM rotation at the end of their fourth year, there is a possibility that student could have taken it twice. Limitations in the NBME scaling process itself may affect the EM-ACE scores observed. In the initial offering of the exam, a “reference group” of fourth-year medical students from LCME-accredited medical schools who were taking the EM-ACE for the first time was used to scale the exam. By definition, incoming interns differ from the reference population at least by level of training, and may differ from the reference group even further if they had taken the EM-ACE before, or attended a non-LCME-accredited medical school. Finally, there was significant colinearity observed among Step 1, Step 2, the EM-ACE and the ABEM ITE. This is likely due to the fact that students who perform well (or poorly) on standardized testing will perform well (or poorly) on all standardized tests. Standardized tests, and student/resident performance on them, do not necessarily assess competency. However, as board certification in our specialty hinges on ABEM qualifying exam performance, standardized examination performance is a proxy measure that holds value.

## CONCLUSION

Performance on the EM-ACE given at the onset of residency correlates well with intern ABEM ITE performance. Earlier assessment of residents’ fund of knowledge may provide program directors with an opportunity for earlier identification of residents with knowledge gaps and increased time to formulate specialized learning plans.

## Figures and Tables

**Table 1:** Demographics of interns completing the emergency medicine advanced clinical examination (EM-ACE) and the in-training exam.

Demographics	Value
Gender, male	58.1%
Age, years	30.4 (4.3)
USMLE, step 1 (n=50)	225 (18.0)
USMLE, step 2 (n=48)	237.7 (17.8)
Weeks of EM prior to in-training	17.6 (4.4)
EM-ACE (scaled) (n=60)	69.8 (7.1)
EM in-training (n=62)	70.5% (8.4%)

*USMLE,* united states medical licensing examination

**Table 2:** Pearson’s correlation coefficients between the United States medical licensing examination (USMLE) Step 1 and Step 2, the emergency medicine advanced clinical exam (EM-ACE) given in July of EM internship and the internship EM in-training exam score.

Exam	USMLE Step 1	USMLE Step 2	EM-ACE	In-training exam
USMLE step 1	N/A	0.70	0.60	0.62
USMLE step 2	0.70	N/A	0.64	0.58
EM-ACE	0.60	0.64	N/A	0.62
In-training exam	0.62	0.58	0.62	N/A
